# The Effects of a 6-Week Controlled, Hypocaloric Ketogenic Diet, With and Without Exogenous Ketone Salts, on Body Composition Responses

**DOI:** 10.3389/fnut.2021.618520

**Published:** 2021-03-24

**Authors:** Alex Buga, Madison L. Kackley, Christopher D. Crabtree, Teryn N. Sapper, Lauren Mccabe, Brandon Fell, Rich A. LaFountain, Parker N. Hyde, Emily R. Martini, Jessica Bowman, Yue Pan, Debbie Scandling, Milene L. Brownlow, Annalouise O'Connor, Orlando P. Simonetti, William J. Kraemer, Jeff S. Volek

**Affiliations:** ^1^Department of Human Sciences, The Ohio State University, Columbus, OH, United States; ^2^Department of Radiology, Davis Heart & Lung Research Institute, The Ohio State University, Columbus, OH, United States; ^3^Division of Cardiovascular Medicine, Department of Internal Medicine, The Ohio State University, Columbus, OH, United States; ^4^Department of Radiology, Wexner Medical Center, The Ohio State University, Columbus, OH, United States; ^5^Research and Development Department, Metagenics, Inc., Aliso Viejo, CA, United States

**Keywords:** ketogenic diet, obesity, exogenous ketones, body composition, advanced imaging

## Abstract

**Background:** Ketogenic diets (**KDs**) that elevate beta-hydroxybutyrate (**BHB**) promote weight and fat loss. Exogenous ketones, such as ketone salts (**KS**), also elevate BHB concentrations with the potential to protect against muscle loss during caloric restriction. Whether augmenting ketosis with KS impacts body composition responses to a well-formulated KD remains unknown.

**Purpose:** To explore the effects of energy-matched, hypocaloric KD feeding (<50 g carbohydrates/day; 1.5 g/kg/day protein), with and without the inclusion of KS, on weight loss and body composition responses.

**Methods:** Overweight and obese adults were provided a precisely defined hypocaloric KD (~75% of energy expenditure) for 6 weeks. In a double-blind manner, subjects were randomly assigned to receive ~24 g/day of a racemic BHB-salt (KD + KS; *n* = 12) or placebo (KD + PL; *n* = 13). A matched comparison group (*n* = 12) was separately assigned to an isoenergetic/isonitrogenous low-fat diet (LFD). Body composition parameters were assessed by dual x-ray absorptiometry and magnetic resonance imaging.

**Results:** The KD induced nutritional ketosis (>1.0 mM capillary BHB) throughout the study (*p* < 0.001), with higher fasting concentrations observed in KD + KS than KD + PL for the first 2 weeks (*p* < 0.05). There were decreases in body mass, whole body fat and lean mass, mid-thigh muscle cross-sectional area, and both visceral and subcutaneous adipose tissues (*p* < 0.001), but no group differences between the two KDs or with the LFD. Urine nitrogen excretion was significantly higher in KD + PL than LFD (*p* < 0.01) and trended higher in KD + PL compared to KD + KS (*p* = 0.076), whereas the nitrogen excretion during KD + KS was similar to LFD (*p* > 0.05).

**Conclusion:** Energy-matched hypocaloric ketogenic diets favorably affected body composition but were not further impacted by administration of an exogenous BHB-salt that augmented ketosis. The trend for less nitrogen loss with the BHB-salt, if manifested over a longer period of time, may contribute to preserved lean mass.

## Introduction

Ketogenic diets (**KDs**) have been demonstrated to promote weight loss, often to a greater extent than low-fat diets, and without explicit instructions to limit calories (1–4). Some authors have suggested that low-carbohydrate diets may facilitate a metabolic advantage by increasing energy expenditure (5), but others have argued against such an effect (6). Irrespective of whether KDs promote greater weight loss than non-KDs, an important unresolved question is how KDs impact the tissue composition and distribution of weight loss, especially when matched for calories. Ideally, weight loss diets promote loss of fat mass (**FM**), especially visceral adipose tissue (**VAT**), while preserving lean tissue.

A plausible approach to support muscle anabolism during a hypocaloric KD is the use of exogenous ketone salts (**KS**), which enhance ketosis while also providing additional sodium and potentially other minerals (e.g., potassium, calcium, magnesium). Oral ingestion of ketone salts consisting of beta-hydroxybutyrate (**BHB**) and minerals is a safe and effective method of transiently increasing circulating ketones as we (7) and others (8) have shown. Notably, prior studies have demonstrated that ketosis achieved by intravenous infusion of sodium BHB preserves fat free mass (**FFM**) during very low-calorie diets and starvation (9, 10). Sodium BHB infusion to levels within the higher range of nutritional ketosis (i.e., ~2–4 mM) has also been shown to increase skeletal muscle protein synthesis, decrease leucine oxidation, and inhibit protein breakdown (11, 12). There is also evidence that ketone bodies may protect from muscle loss in catabolic settings such as sarcopenia, cachexia, and excessive inflammation (12–15), perhaps through mTORC1 signaling (16, 17). Based on evidence from these prior studies, we hypothesized that augmenting ketosis by oral ingestion of BHB-salts might lead to preservation of muscle during a hypocaloric KD intervention in humans.

Whereas, many studies have demonstrated that short-term hypocaloric, low-carbohydrate diets result in preservation of FFM and preferential loss of fat (18–21), including VAT (4), other studies have reported similar (22, 23) or greater loss of lean body mass in subjects on KDs (24, 25). Several factors may explain the discrepant results on the composition of weight loss to KDs. First, consuming protein at a level <1 g/kg ideal body mass impairs nitrogen balance while an intake closer to 1.5 g/kg ideal body mass appears optimal during hypocaloric diets for maintaining lean mass (20, 24, 26). Second, many KD studies failed to provide adequate sodium to compensate for the natriuretic effect of these diets, resulting in counter-regulatory hormonal responses that adversely impacts sodium and potassium balance (23). In the KD studies that did not provide adequate sodium, nitrogen balance, and lean mass were compromised (24, 25). Lastly, common methods of assessing body composition, such as hydro-densitometry and dual-energy x-ray absorptiometry (**DXA**), detect water loss as a decrease in lean tissue, but this does not reflect a change in whole-body intracellular protein. For example, reduced extra-vascular volume and intra-cellular glycogen (~3 g of water is stored with each gram of glycogen), which can easily account for 2–3 kg during the first few weeks of a KD (22, 27), would be artifactually detected as a loss of lean tissue.

Collectively, these data highlight a potential role of well-formulated KDs with adequate protein and sodium to improve preservation and lean body mass during weight loss, with potential added benefit of exogenous ketones. Using a randomized, controlled-feeding, double-blind design, the primary objective of this study was to explore the composition of weight loss, including advanced imaging assessment of fat and lean mass as well as nitrogen excretion and 3-methylhistidine (**3MH**), to a well-formulated KD with and without KS supplementation. After completing this primary objective, we decided it would be valuable to compare these KD group responses to a matched group of obese adults fed a hypocaloric low-fat diet. We used DXA to assess whole body adiposity and lean mass, magnetic resonance imaging (**MRI**) to assess VAT mass and thigh skeletal muscle cross-sectional areas, as well as nitrogen balance and 3MH to provide additional measures of protein metabolism.

## Materials and Methods

### Experimental Design and Subjects

This was a prospective, placebo-controlled, double-blind study design. For the primary aim, we planned to randomize and balance *n* = 28 (14 men/14 women) in two KD groups. Eligibility was determined based on BMI (27–35 kg/m^2^) and age (21–65 years). Twenty-eight participants were consented and 25 completers were analyzed. We performed a stratified randomization where “age” and “BMI” were divided “below” or “above” the median range of the inclusion criteria. Following stratification, we used an online number generator (www.randomizer.org) to randomize eight rounds of three participants to either a ketogenic diet + ketone supplement (**KD + KS**, *n* = 12, 6 men/6 women) or ketogenic diet + placebo (**KD + PL**, *n* = 13, 6 men/7 women). An extra male participant was added to KD + PL due to enrolment errors. For the secondary aim, we enrolled a separate, non-randomized group of age and BMI-matched participants who received an isoenergetic/isonitrogenous low-fat diet (**LFD**, *n* = 12, 6 men/6 women) and participated in the same testing as the KD groups.

Exclusion criteria comprised: major weight loss events (<10% body mass) 6 months prior to enrollment; habitually consuming a low-carbohydrate diet (<50 g CHO/day); pre-existing gastrointestinal disorders or food allergies; excess alcohol consumption (>14 drinks/week); disease conditions (diabetes, liver, kidney, or other metabolic or endocrine dysfunction); and use of diabetic medications. Participants who met the qualifying criteria were scheduled for an in-person screening meeting where the study was described in greater detail followed by completing questionnaires about food frequency, medical history, physical activity, MRI readiness, and a menstrual history survey. There were no significant differences in baseline characteristics of completers between groups (Table 1). All participants were Caucasian. For added clarity a CONSORT diagram is available in (Supplementary Figure 1).

**Table 1:** Baseline characteristics.

**Variable**	**KD + KS** **(6M/6F)**	**KD + PL** **(6M/7F)**	**LFD** **(6M/6F)**	***p*-value**
Age (years)	35 ± 3	35 ± 3	35 ± 3	0.99
Height (cm)	171.5 ± 2.9	172.1 ± 2.7	172.6 ± 2.9	0.96
Weight (kg)	90.4 ± 3.4	94.1 ± 3.2	92.4 ± 3.4	0.73
BMI (kg/m^2^)	30.6 ± 0.7	31.8 ± 0.7	30.9 ± 0.7	0.50
Waist Circumference (cm)	96 ± 3	95 ± 2	92 ± 3	0.66
Hip Circumference (cm)	109 ± 2	114 ± 2	111 ± 2	0.14
Lean Mass (kg)	55.8 ± 3.1	55.3 ± 3.0	56.0 ± 3.1	0.99
Fat Mass (kg)	31.1 ± 2.2	34.5 ± 2.1	33.4 ± 2.2	0.52
Body Fat Percentage (%)	35%±2%	38%±2%	36%±2%	0.68
Capillary β-hydroxybutyrate (mmol/L)	0.18 ± 0.03	0.18 ± 0.04	0.13 ± 0.02	0.40

Values reported as mean ± SEM. p-value obtained from one-way ANOVA.

BMI, body mass index; KD, ketogenic diet; KS, ketone salts; PL, placebo; LFD, low-fat diet; M/F, men/women randomized to each diet.

### Feeding Intervention

All meals for the three experimental diets were prepared in a state-of-the-art metabolic kitchen. All the ingredients were precisely weighed (±0.1 g) by research staff. Individual menu composition had custom macro- and micro-nutrients calculated by a team of registered dietitians using advanced nutrient analysis software (Table 2) (Nutritionist Pro, Axxya Systems, Redmond, WA). Both KDs were designed based on our previous work (28, 29). The LFD was developed in accordance with the USDA's Dietary Guidelines for Healthy Americans 2015–2020 (30). Ideal body weight was calculated based on the Metropolitan Life Tables for medium frame populations to establish a 1.5 g protein/kg of ideal body weight for each participant (31). The total daily energy required for weight-maintenance was derived from Harris-Benedict basal metabolic rate multiplied by physical activity level (PAL) (32). PAL was determined based on previous guidelines for sedentary individuals created by the Institute of Medicine (33). Average PAL multiplier was estimated at 1.33 (range: 1.20–1.55). The individual energy needs to achieve weight loss were calculated as 75% of estimated energy requirements for weight maintenance, which provided on average 20 kcal/kg/day (range: 16–26 kcal/kg/day).

**Table 2:** Diet composition.

	**KD + KS**	**KD + PL**	**LFD**
Energy (kcal/day)	1845 ± 102	1752 ± 98	1900 ± 102
Protein (g)	99 ± 3	100 ± 3	100 ± 3
Carbohydrate (g)	40 ± 8^a^	38 ± 7^a^	259 ± 8^b^
Sugar (g)	17 ± 3^a^	17 ± 3^a^	101 ± 3^b^
Fiber (g)	10 ± 1^a^	10 ± 1^a^	34 ± 1^b^
Added sugars (g)	n/a	n/a	<25 g/day
Fat (g)	143 ± 9^a^	131 ± 8^a^	51 ± 9^b^
SFA (g)	63 ± 4^a^	63 ± 4^a^	17 ± 4^b^
MUFA (g)	38 ± 3^a^	38 ± 3^a^	10 ± 3^b^
PUFA (g)	8 ± 1	8 ± 1	7 ± 1
Cholesterol (g)	414 ± 27^a^	402 ± 26^a^	154 ± 27^b^
Sodium (mg)	6100 ± 32^a^	2351 ± 30^b^	1974 ± 31^c^
Potassium (mg)	2211 ± 73^a^	2243 ± 75^a^	2758 ± 78^b^
Calcium (mg)	2001 ± 36^a^	880 ± 34^b^	1008 ± 35^c^

Values reported as mean ± SEM. Distinct letters denote group differences (p < 0.05).

SFA, saturated fatty acids; MUFA, monounsaturated fatty acids; PUFA, polyunsaturated fatty acids.

Both KDs provided ~40 g/day of carbohydrates and the remaining non-protein calories were derived from fat, with an emphasis on monounsaturated and saturated fat sources. The LFD provided 25% of energy from lipids with <10% saturated fat and <30 g added oils. Carbohydrates were primarily complex and provided at least 32 g/fiber per day with limited and added sugars (<25 g). *Ad libitum* intake of calorie-free/sodium-free products was allowed during the entire intervention. A variety of whole foods were used to develop both ketogenic and low-fat meals. Meal plans were developed to include a wide range of high-quality protein sources to be distributed equally between breakfast, lunch, and dinner (Supplementary Table 1) with minor differences in total amino acid (AA) intake, particularly histidine (LFD vs. KD: −520 ± 20 mg/day). Because the LFD was designed in accordance with Dietary Guidelines for Americans, a minor percentage of the total protein was derived from plant sources such as quinoa, whole grain pasta/rice/bread, oatmeal, legumes (8–10%), compared to predominantly animal protein in the KD. A portion of the protein for the KD groups was provided as two daily chocolate or vanilla shakes that contained whey protein isolate (~15 g/serving) along with fat in the form of high oleic sunflower oil and medium chain fatty acids (MCT). Assessment of capillary glycemic response over 2 h following the ketogenic shake in healthy participants (*n* = 10) compared with a white bread control matched for total carbohydrate content (9 g), demonstrated that there was no glycemic response to these shakes (Supplementary Figure 2). In addition, subjects in the KD groups consumed one serving of 10 g MCT oil (caprylic and capric acid; Metagenics, Inc) with their breakfast and one serving in the afternoon snack.

### Supplement

The KD + KS group consumed a ketone supplement, twice daily, consisting of BHB salts and non-caloric flavoring (provided by Metagenics, Inc., Aliso Viejo, CA). This particular KS has been previously reported to raise BHB 4 to 5-fold in capillary blood of non-keto adapted individuals, with significant effects observed up to 1-h post-ingestion (34). One KS serving contained 11.8 g BHB, 1,874 mg sodium, 570 mg calcium, and 57 mg magnesium. BHB content was determined in our laboratory to contain a racemic BHB enantiomer mixture of R-BHB and S-BHB. Participants in the KD-KS group were instructed to mix the KS in at least 250 ml of water and stir vigorously to ensure proper mixing. One dose was taken in the morning and one 6h later after lunch. Because the ketone supplement contained calories in the form of BHB, the fat content of the KD + KS group was reduced by ~120 kcal/day to ensure that all three diets were isocaloric. The KD + PL group and the LFD received a calorie-free flavored placebo that contained no BHB or minerals. The placebo was specifically formulated by the sponsor to mimic the taste and appearance of active supplement including flavoring but contain no BHB-salts. The same placebo was used in a previous trial with no blinding problems (33). In our pilot testing with the BHB salt we compared the supplement to the placebo in a double-blind setting with members of the group who were not involved in data collection and analysis (*n* = 3). Based on their feedback there were no discernable traits between the BHB salt and placebo drinks. Based on conversations with participants throughout the study, we feel confident that they were not aware which supplement they received.

### Lab Procedures

A battery of tests were performed biweekly at WK0 (baseline), WK2, WK4, and WK6. All participants reported to the testing facility between 5:00 and 7:00 a.m. for their assessments (PAES Building, 305 Annie and John Glenn Ave, Columbus, OH). Arrival conditions stipulated that subjects consume no caffeine for >12 h, no food for >8 h, sleep 8–10 h the night before testing, and abstain from strenuous exercise <48 h prior to the visit. Weight and height were measured on an electronic stadiometer (SECA 703 Digital, Hamburg, Germany) calibrated to the nearest ±0.1 cm and ±0.01 kg, respectively, while participants wore light clothing and no shoes. Urine specific gravity was measured with a light refractometer (Reichert™, Buffalo, NY). If values were >1.025, the participant was instructed to drink at least 250 ml of water until euhydration was attained. Waist and hip measurements were performed by the same investigator using a manual tape (Gulick Spring Tape) to measure circumference at the narrowest part of the waist and the greater trochanter region according to previous testing guidelines (35). Resting energy expenditure (REE) and respiratory exchange ratio (RER) were measured *via* indirect calorimetry (ParvoMedics TrueOne® 2,400) (36). After a 20 min supine rest, gas exchanges were measured for 25 min at 15-s intervals. REE was selected from a stable, average interval recorded within the last 5 min of continuous readings to avoid artificial number inflations caused by early REE fluctuations during calibration.

Fasting BHB concentrations were assessed in capillary blood using reagent strips and a monitoring device (Abbot FreeStyle®, Columbus, OH). Outside of test days, participants reported fasted ketones every morning during the study *via* image texts sent to research staff. Fasting venous blood samples were collected on testing days *via* venipuncture in the antecubital fossa. A single plasma tube (10ml) was inverted and spun immediately after blood draw at 1200 × G for 10 min at 4°C. Plasma fractions were aliquoted in screwcap vial containers, snap frozen in liquid nitrogen, and stored at −80°C for subsequent analysis. Plasma 3MH was assayed in duplicate using a commercially available enzyme-linked immunosorbent assay kit (Wuhan Fine Biotech). Intra- and inter-assay coefficients of variation were 4.3 and 6.8%, respectively.

For urine collection, participants were provided with a 3.7-L rated container to void their 24 h urine after each biweekly visit. Of note, the first 24 h urine collection was not a true baseline because it occurred during day one of the KD and KS intervention. Thus, the baseline 24 h period included initial exposure to ketogenic meals as well as two doses of the KS or PL. The containers were pre-treated with a stabilizer (Gentamicin 20 mg, Germall II 1.25 g) to prevent metabolite degradation and disappearance at room temperature. All urine samples were returned immediately after the 24 h period, the total volume recorded, and aliquots stored at −20°C before shipping to an off-campus collaborator (Litholink Corporation, Chicago, IL, USA). Urine creatinine and urea nitrogen were measured on a Beckman AU 680 autoanalyzer (Beckman Instruments, Brea CA); creatinine by a kinetic alkaline picrate method and urea nitrogen by an enzymatic method incorporating urease and glutamate dehydrogenase. Urine urea nitrogen (**UUN**) was standardized to creatinine excretion. Nitrogen balance was calculated using a previously validated formula (37):

$$\begin{array}{l} \text{Nitrogen Balance}=\text{Protein Intake}\;(\text{g}/\text{day})\;*\;0.16\\ \;-\text{Urine Urea Nitrogen}\;(\text{g}/\text{day})+4 \end{array}$$

### Body Composition

FM and FFM were quantified by dual X-ray absorptiometry (DXA) on a Lunar iDXA system (GE, Madison, WI, USA). The DXA was quality assured by a licensed technician every 2 days to ensure accuracy. Subjects were carefully aligned within detection limits on the DXA bed. The average duration of a whole-body scan lasted 7 min. A single, whole-body, DXA measurement was performed on each subject in order to estimate lean and fat mass (CoreScan^TM^ enCORE software version 14.10) using two-dimensional projection data created by low energy, fan beam x-ray to create a model consisting of bone, adipose, and lean tissue compartments.

We used MRI to obtain a more precise measure of VAT, subcutaneous adipose tissue (**SAT**) and mid-thigh muscle cross-sectional area (**CSA**). Each participant was imaged once at baseline and once post-intervention on a 3T scanner (MAGNETOM Prisma Fit, Siemens Healthineers, Erlangen, Germany). The VARiable PROjection (VARPRO) pulse sequence was used to acquire the in-phase, out-phase, water, water percentage, fat, and fat percentage images that were used to measure abdominal fat volumes. The VARPRO pulse sequence is a single breath hold acquisition that collects the multiple echo time images required for fat/water separation. This rapid scan technique acquires 3D volumetric images covering the entire abdominal region in a single breath-hold. The total duration of the testing session averaged 45 min to 1 h.

Analysis of abdominal fat was performed using a custom built, previously described MatLab algorithm (38) using semi-automated segmentation and calculation of VAT and SAT across the full abdomen defined as 20% of the distance from the top of the iliac crests to the base of the skull. Mid-thigh CSA was measured as a single slice at the mid-point between the inguinal crease and the proximal patella. Based on previous studies that have validated this site-specific technique in sarcopenia or muscular atrophy (39, 40) we used MRI mid-thigh CSA as a specific measure of muscle mass to complement whole-body FFM measures detected by DXA. Manual traces were drawn in the anterior, posterior, and medial compartment of the thigh then combined to obtain a whole muscle CSA value. The contours were drawn so as to avoid inter-muscular fat and artifacts using ImageJ (41).

### Statistics

The sample size chosen was based on what has been done in similar feeding studies (21, 23–25, 27) and what was feasible to accomplish based on the available funding and resources. Analyses were performed using SPSS Ver. 25 (SPSS, Inc., Chicago, IL, USA). Two-tail α significance was set at *p* < 0.05. Baseline measures were compared for significant differences with one-way analysis of variance (ANOVA). We considered this an exploratory study and therefore analyzed main effects and interactions between KDs using a 2 (group) × 4 (time) repeated measures ANOVA without adjusting for multiple comparisons. To provide additional perspective, we also explored the comparison between the two randomized KD groups and the non-randomized LFD group in a 3 × 4 ANOVA. Independent *t*-tests were utilized for the pre-post MRI variables. Due to baseline variability and differences in histidine intakes, we subtracted the individual 3-methylhistidine values from their respective baseline (normalized values) and analyzed the normalized means for main effects and interactions using baseline as covariate. Fisher's Least Significant Difference (LSD) correction was used for all *post-hoc* analyses. All data are presented as mean ± SEM.

## Results

### KD + KS vs. KD + PL

**Figure 1:**
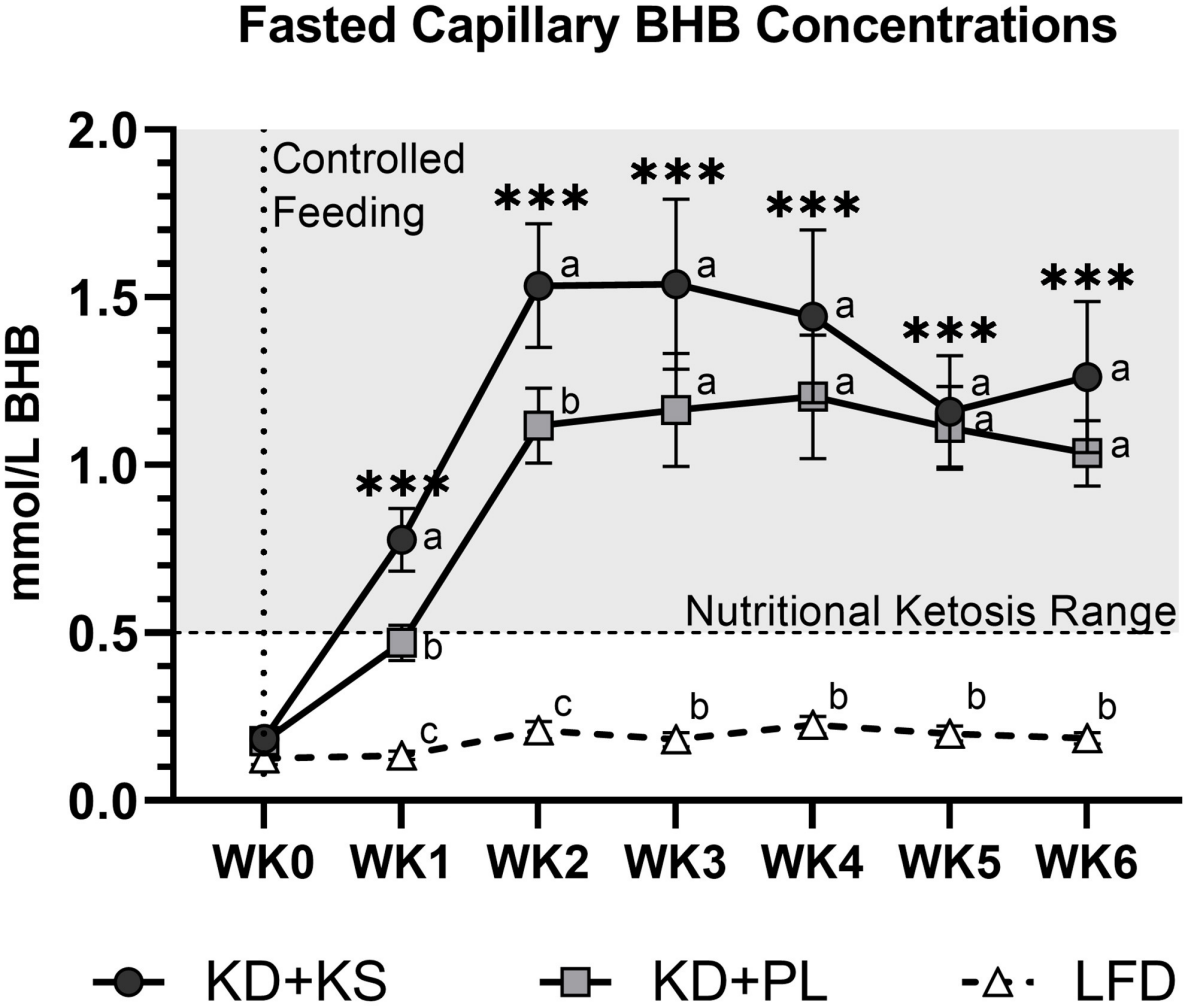
Fasting capillary BHB responses to a ketogenic diet plus ketone salt (KD + KS) vs. placebo (KD + PL). Values at each week represent mean (± SEM) of 7-day BHB values. Primary Statistics: 2 (group) × 7 (time point) ANOVA followed by secondary 3×7 ANOVA that included a third low-fat diet (LFD) group (dashed line). Time effects: ***p < 0.001 from WK0 for primary statistics only. Interaction effects: distinct letters at each time point denote between group differences (*p* < 0.05).

In both KD groups, fasted capillary BHB increased progressively to concentrations above 1.0 mmol/L by WK2 and stayed elevated throughout the 6-week intervention (Figure 1). Fasting BHB was higher in KD + KS at WK1 and WK2 but was similar to KD + PL thereafter. REE decreased at WK2 and stayed lower throughout the intervention with no differences between groups. RER decreased at WK2 and remained lower throughout the intervention (Table 3).

**Table 3:** Diet effects and interactions.

**Variable**	**Diet**	**Timepoint**				**Change**		**ES**	**2x4 ANOVA (*****p*****-value)**		
		**WK0**	**WK2**	**WK4**	**WK6**	**Δ**	**% Δ**	***d***	**Group**	**Time**	**Interaction**
			***	***	***						
Weight (kg)	KD + KS	90.4 ± 3.4	86.6 ± 3.3	84.6 ± 3.3	83.1 ± 3.3	−7.3	−8%	0.35	0.47	**<0.001**	0.45
	KD + PL	94.1 ± 3.2	89.5 ± 3.2	87.9 ± 3.2	86.1 ± 3.2	−8.0	−9%				
			***	***	***						
BMI (kg/m^2^)	KD + KS	30.6 ± 0.7	29.4 ± 0.7	28.7 ± 0.7	28.2 ± 0.8	−2.4	−8%	0.36	0.34	**<0.001**	0.49
	KD + PL	31.8 ± 0.7	30.3 ± 0.7	29.7 ± 0.7	29.1 ± 0.7	−2.6	−8%				
			***	***	***						
Waist circumference (cm)	KD + KS	95.7 ± 2.5	93.5 ± 2.7	91.0 ± 2.5	87.7 ± 2.6	−8.0	−8%	0.20	0.65	**<0.001**	0.32
	KD + PL	94.7 ± 2.4	91.0 ± 2.6	89.0 ± 2.4	87.4 ± 2.5	−7.3	−8%				
			***	***	***						
Hip circumference (cm)	KD + KS	108.6 ± 1.8	106.7 ± 1.7	103.9 ± 1.9	103.8 ± 1.8	−4.7	−4%	0.61	0.06	**<0.001**	0.26
	KD + PL	113.7 ± 1.8	110.8 ± 1.7	109.0 ± 1.8	107.5 ± 1.8	−6.3	−6%				
					***						
Waist: hip ratio (cm/cm)	KD + KS	0.88 ± 0.03	0.88 ± 0.02	0.88 ± 0.02	0.85 ± 0.02	−0.04	−4%	0.38	0.13	**<0.001**	0.23
	KD + PL	0.84 ± 0.02	0.82 ± 0.02	0.82 ± 0.02	0.81 ± 0.02	−0.02	−3%				
			***	***	***						
Lean body mass (kg)	KD + KS	55.8 ± 3.1	54.2 ± 3.1	54.2 ± 3.0	53.9 ± 3.1	−1.9	−3%	0.04	0.91	**<0.001**	0.92
	KD + PL	55.3 ± 3.0	53.9 ± 3.0	53.6 ± 2.9	53.3 ± 2.9	−1.9	−4%				
			***	***	***						
Body fat mass (kg)	KD + KS	31.1 ± 2.2	29.4 ± 2.1	27.7 ± 2.1	26.4 ± 2.1	−4.8	−15%	0.24	0.20	**<0.001**	0.63
	KD + PL	34.5 ± 2.1	32.8 ± 2.0	31.4 ± 2.0	30.2 ± 2.1	−4.4	−13%				
			**	***	***						
Lean: fat mass ratio (kg/kg)	KD + KS	1.9 ± 0.2	1.9 ± 0.3	2.0 ± 0.3	2.1 ± 0.3	0.3	15%	0.31	0.56	**<0.001**	0.44
	KD + PL	1.7 ± 0.2	1.8 ± 0.2	1.8 ± 0.3	1.9 ± 0.3	0.2	13%				
			*	***	***						
Body fat percentage (%)	KD + KS	35.0 ± 2.2	34.6 ± 2.2	33.2 ± 2.3	32.2 ± 2.3	−2.8		0.08	0.41	**<0.001**	0.68
	KD + PL	37.7 ± 2.1	36.9 ± 2.1	35.9 ± 2.2	35.0 ± 2.2	−2.7					
					***						
Visceral adipose tissue (g)	KD + KS	2978 ± 589			2378 ± 520	−600	−20%	0.24	0.98	**<0.001**	0.56
	KD + PL	2947 ± 542			2434 ± 479	−513	−17%				
					***						
Subcutaneous adipose tissue (g)	KD + KS	5220 ± 639			4282 ± 539	−938	−18%	0.05	0.44	**<0.001**	0.90
	KD + PL	5869 ± 588			4894 ± 496	−974	−17%				
			***	***	***						
Resting energy expenditure (kcal/day)	KD + KS	1885 ± 93	1774 ± 86	1621 ± 82	1653 ± 87	−231	−12%	0.35	0.41	**<0.001**	0.30
	KD + PL	1739 ± 90	1605 ± 83	1609 ± 79	1604 ± 84	−135	−8%				
			***	***	***						
Respiratory exchange ratio (V_CO2_/V_O2_)	KD + KS	0.83 ± 0.02	0.75 ± 0.01	0.76 ± 0.01	0.77 ± 0.01	−0.07	−8%	0.28	0.06	**<0.001**	0.69
	KD + PL	0.86 ± 0.02	0.78 ± 0.01	0.77 ± 0.01	0.78 ± 0.01	−0.08	−10%				
Urea nitrogen (g)	KD + KS	7.4 ± 0.3	7.8 ± 0.4	7.1 ± 0.3	6.7 ± 0.3	−0.7	−9%	0.12	0.08	**0.014**	0.98
	KD + PL	7.8 ± 0.3	8.3 ± 0.4	7.8 ± 0.3	7.3 ± 0.3	−0.5	−6%				
Nitrogen balance	KD + KS	4.5 ± 0.6	4.0 ± 0.7	4.7 ± 0.6	5.2 ± 0.6	0.7	16%	0.12	0.58	**0.014**	0.99
	KD + PL	4.1 ± 0.5	3.6 ± 0.7	4.2 ± 0.6	4.6 ± 0.6	0.5	12%				
3-Methylhistidine (nmol/mL)	KD + KS	43 ± 8	53 ± 21	31 ± 13	46 ± 14	4	8%	0.51	0.29	0.19	0.46
	KD + PL	24 ± 3	39 ± 11	34 ± 5	51 ± 10	27	110%				

Values reported as mean ± SEM. Time effects:

* p < 0.05;

** p < 0.01;

*** p < 0.001 compared to WK0.

*Δ = absolute change from WK0. %Δ = percent change from WK0. ES = effect size (Cohen's d). Bold face higlights significant effects*.

**Figure 2:**
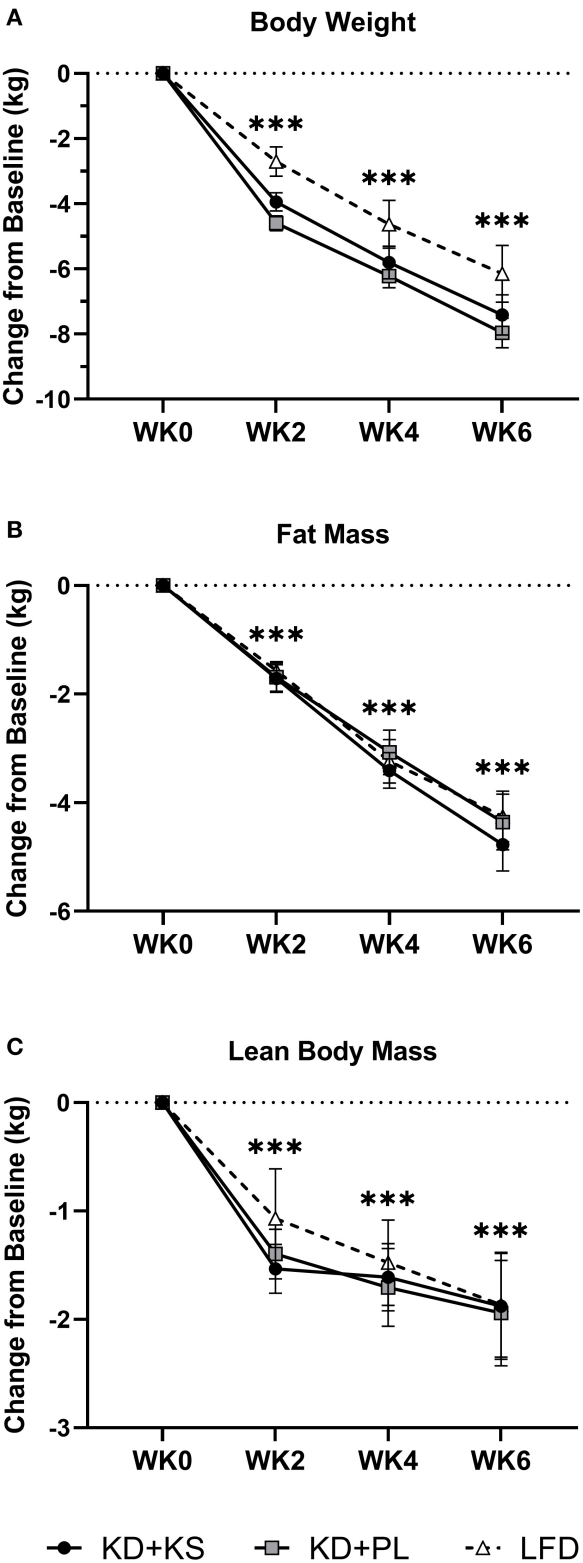
Diet effects on body weight and weight loss composition. Solid lines are primary KD outcomes; dashed lines show LFD for comparison. Results are presented as absolute change (± SEM) from baseline. Panels: **(A)** scale body weight, **(B)** DXA whole-body fat and **(C)** DXA whole-body lean mass. Statistics: 2 (group) × 4 (time) ANOVA. Time effects: ***p < 0.001 from WK0. Body weight and fat mass decreased steadily and significantly between each timepoint after WK0 (*p* < 0.001). Lean mass decreased significantly by WK2 (*p* < 0.001), followed by no other significant reductions thereafter. With LFD included in the secondary 3 (group) × 4 (time) ANOVA analysis – No major changes were detected besides a higher FFM WK2 value compared to WK6 (*p* < 0.05).

There were significant changes over time in all DXA and MRI parameters of body composition, but no group differences (Figures 2A–C). Body mass, FM, and FFM decreased at the end of the feeding intervention by −7.7 ± 0.4, −4.6 ± 0.4, and −1.9 ± 0.3 kg, respectively (*p* < 0.001). The trajectory of body weight loss was most pronounced in the first 2 weeks (−4.6 ± 0.2% BW) and accounted for more than half (57%) of the total body weight that was lost during the intervention. Additionally, more than three-quarters of lean mass were lost by WK2 (77%) and continued toward a non-significant decrease thereafter. MRI analysis indicated a significant loss of both VAT (−19 ± 2 %; *p* < 0.001) and SAT (−17 ± 3%; *p* < 0.001). The whole-body lean-to-fat mass ratio improved in both KD groups (+0.3 ± 0.1 kg/kg; *p* < 0.001), showing a preference in body fat reduction over muscle. Mid-thigh whole quadriceps muscle CSA decreased over time (−5 ± 1%; *p* < 0.001) and corroborated the trends seen in DXA (Table 4). Waist circumference (WC) decreased in KD + KS (−8.0 ± 1.0 cm; *p* < 0.001) and KD + PL (−7.3 ± 1.0 cm; *p* < 0.001) starting at WK2 and continuing through WK6. Waist-to-hip ratio (WHR) also decreased in both KD + KS (−0.04 ± 0.01; *p* < 0.001) and KD + PL (−0.02 ± 0.01; *p* < 0.05) by WK6 (Table 3).

**Table 4:** Mid-thigh cross-sectional area.

**Variable**	**Diet**	**Timepoint**		**Change**		**ES**	**2 x 2 ANOVA (*****p*****-value)**		
		**WK0**	**WK6**	**Δ**	**%Δ**	***d***	**Group**	**Time**	**Interaction**
			***						
ANTERIOR (mm^2^)	KD + KS	7599 ± 543	7340 ± 511	−259	−3%	0.03	0.22	**<0.001**	0.94
	KD + PL	6717 ± 522	6450 ± 491	−267	−4%				
			***						
POSTERIOR (mm^2^)	KD + KS	3527 ± 205	3361 ± 183	−166	−5%	0.47	0.26	**<0.001**	0.25
	KD + PL	3257 ± 197	2986 ± 176	−272	−8%				
			***						
MEDIAL (mm^2^)	KD + KS	3675 ± 322	3462 ± 315	−213	−6%	0.06	0.40	**<0.001**	0.88
	KD + PL	3385 ± 309	3154 ± 303	−230	−7%				
			***						
WHOLE (mm^2^)	KD + KS	14801 ± 961	14163 ± 893	−638	−4%	0.22	0.21	**<0.001**	0.58
	KD + PL	13359 ± 923	12590 ± 858	−769	−6%				

Values reported as mean ± SEM. ES = effect size (Cohen's d).

Time effects:

*** p < 0.001 compared to WK0. Δ = absolute change from WK0. %Δ = percent change from WK0.

*Anterior compartment: quadriceps (vastus lateralis/medialis, rectus, sartorius); Posterior compartment: biceps femoris, semimembranosus, semitendinosus; Medial compartment: adductor longus/magnus, gracilis. Bold face higlights significant effects*.

**Figure 3:**
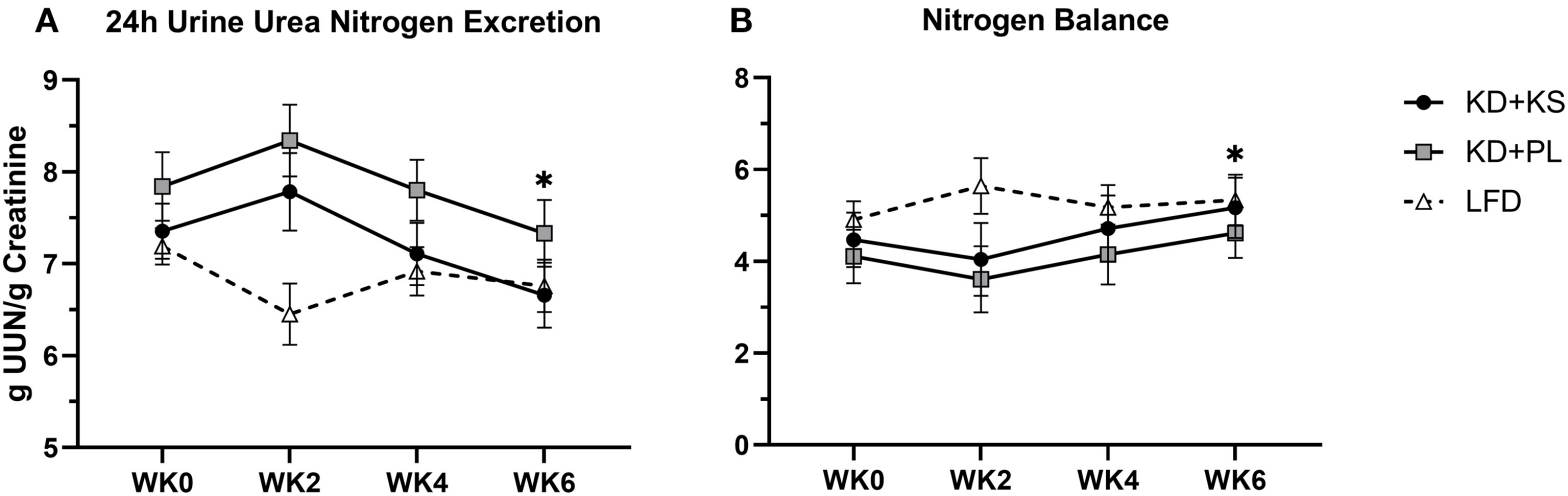
Diet effects on urinary nitrogen and nitrogen balance. Solid lines are primary KD outcomes; dashed lines show LFD for comparison. Panels: **(A)** 24 h urine urea nitrogen standardized per gram of creatinine; **(B)** nitrogen balance derived from validated formula. Primary analysis: 2 (group) × 4 (time) ANOVA. WK2 UUN was significantly higher than WK4 (*p* < 0.05) and WK6 (*p* < 0.01). Group effects show on average 8% lower UUN during KD + KS compared to KD + PL (*p* = 0.082). With LFD included in the secondary 3 (group) × 4 (time) ANOVA analysis – Time effects: *p < 0.05 from WK0. Between-group effects: KD + PL had significantly higher UUN than LFD (*p* = 0.004) and trended higher than KD + KS (*p* = 0.076).

**Figure 4:**
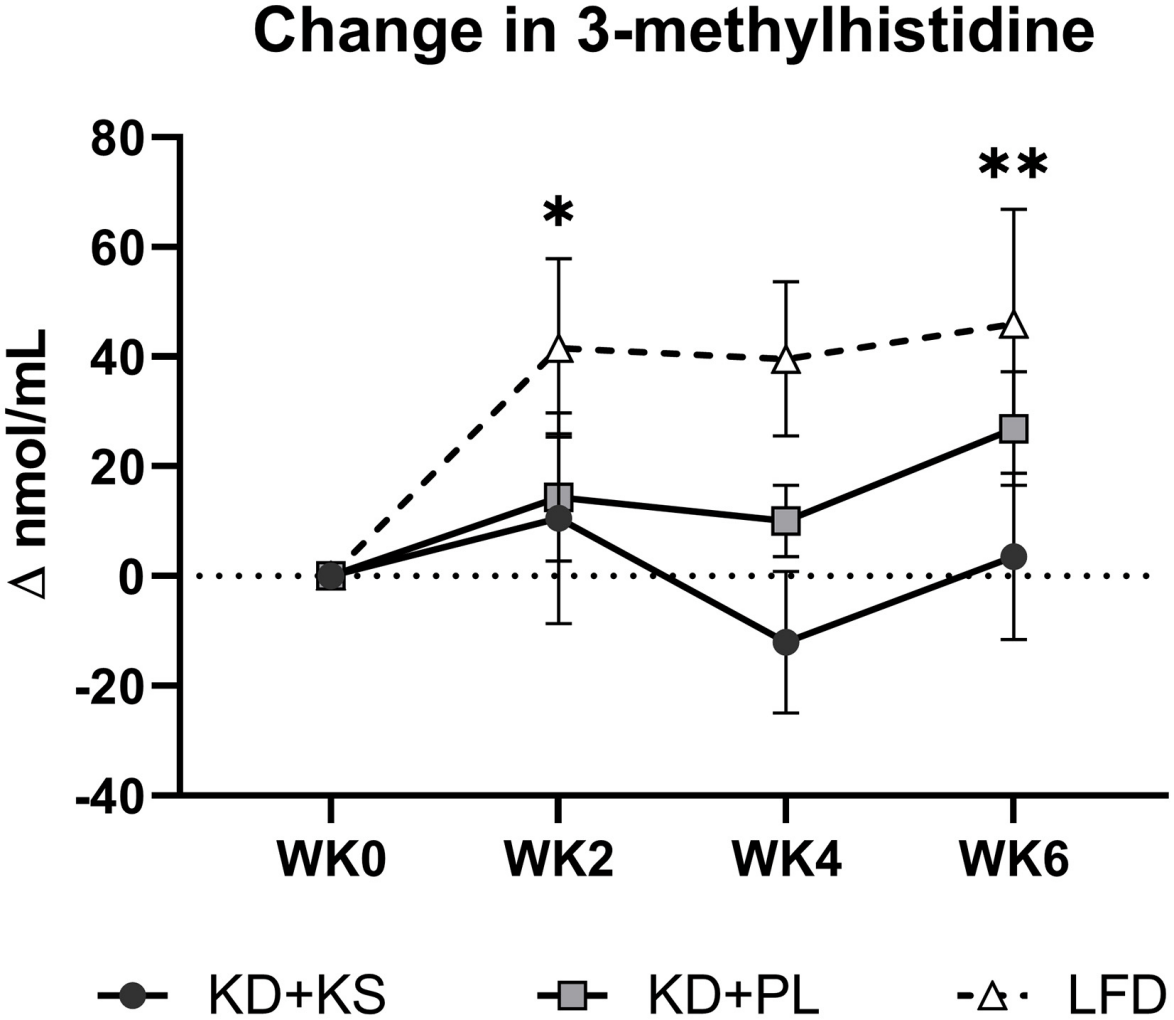
Change in normalized plasma 3-methylhistidine relative to baseline. Values were subtracted from WK0 plasma 3MH concentrations and analyzed for significant covariance effects. Primary statistic: 2 (group) × 4 (time) ANOVA with normalized baseline held as covariate. No significant effects were detected between KD groups. With LFD included in the secondary 3 (group) × 4 (time) ANOVA – Time effect: *p < 0.05; **p < 0.01 from WK0.

Concentrations of UUN demonstrated a slight increase at WK2 and then declined significantly from that point at WK4 (*p* < 0.05) and WK6 (*p* < 0.01). There was a group trend for lower UUN excretion in the KD + KS group (−8%, *p* = 0.082) (Figure 3A). Nitrogen balance (NB) demonstrated a minor decrease at WK2 that increased significantly from that point at WK4 (*p* < 0.05) and WK6 (*p* < 0.01), but there were no significant group trends (*p* = 0.58) (Figure 3B). Normalized plasma 3MH values did not change significantly (Figure 4).

### KD + KS vs. KD + PL vs. LFD

For most parameters, the LFD responded in a similar manner as the KDs with a few notable exceptions (Supplementary Table 2). The KDs significantly altered ketones and RER, but these variables were not altered by the LFD. WC reductions were evident only by WK6 on LFD (−2.9 ± 1.0 cm; *p* = 0.008) whereas, WHR was not significantly altered in this group. We discovered a significant interaction for WC and WHR, with both KD groups showing a greater decrease. The main group effect for UUN was significant in the 3 × 4 ANOVA (*p* = 0.015) and revealed that the KD + PL group excreted significantly more urea nitrogen than LFD (*p* = 0.004) (Figure 3A) while trending higher compared to KD + KS (*p* = 0.076). There was a significant time effect for 3MH that indicated higher concentrations at WK2 (*p* < 0.05) and WK6 (*p* < 0.01) compared to baseline (Figure 4).

## Discussion

This study investigated the effects of daily exogenous ketone BHB supplementation (in the form of a racemic BHB salt) in the context of a 6-week energy-controlled ketogenic feeding intervention that produced clinically meaningful weight loss. Although the KS transiently elevated BHB concentrations beyond the KD alone, it did not alter the magnitude of weight loss or changes in whole body and regional tissue composition including VAT, SAT, and thigh muscle CSA. There was a trend for lower UUN with the addition of a KS to the KD, but this was not of sufficient magnitude to manifest in a detectable change in FFM by DXA or muscle CSA by MRI over a 6-week period. The body composition responses to the KDs were similar to those observed after a calorically matched LFD.

As expected, the KD elevated fasting BHB (mean 1.1 mM), which was augmented by addition of the KS (mean 1.4 mM) during the first 2 weeks of the intervention. Ketosis was achieved in as early as 3 days, similar to what we have reported before with implementation of a well-formulated KD (4). It is important to point out that the fasting measures of BHB were all done in the morning after an overnight fast and at least 10 h following the ingestion of the last dose of KS the evening of the previous day. In keto-naïve people (fasting BHB 0.1–0.2 mM), the exact same KS and dose has previously been shown to result in a rapid increase in capillary BHB peaking at 1 mM after approximately 15 min and gradually returning to baseline by 2 h (34). Considering the transient nature of BHB elevation after consumption of the KS, the fact that we were able to detect higher fasting BHB 10–12 h later in the KD + KS group was somewhat unexpected. The augmentation of fasting BHB in response to exogenous ketones only lasted 2 weeks, suggesting that adaptations in the metabolic regulation of ketone homeostasis (e.g., BHB oxidation, renal clearance, etc.) occurred.

We hypothesized that augmentation of ketosis by twice daily ingestion of a KS might enhance preservation of lean mass in response to a hypocaloric KD that produced clinically meaningful weight loss, but results from our 6-week intervention suggest that was not the case. For every kilogram of FFM lost, FM decreased between 2.3 and 2.5 kg, a ratio that is consistent with previously published KD studies (42). These results must be viewed with an understanding that DXA determines fluid loss as a decrease in FFM, and KDs are associated with initial loss of body water (22, 43). KDs have been shown to acutely reduce glycogen stores by 50% (44), which could account for nearly 1 kg of lean tissue. KDs are also associated with a natriuretic and diuretic effect that could lead to greater loss of fluid, especially if not counter-acted by increased sodium and fluid intake, again being reflected by increased loss of lean tissue. It is notable that the two KDs contained significantly different amounts of sodium due to the high sodium content of the KS (KD + PL = 2,351, KD + KS = 6,100 mg/day), yet this did not impact DXA determined FFM responses. Furthermore, whole body FFM analyses can be affected by the trunk area due to the presence of confounding organs in the region of interest, so appendicular regions, such as the thigh, have been used to circumvent this limitation in FFM assessment (45). Given these limitations of DXA we also used MRI assessment of thigh musculature, which demonstrated significant CSA reductions after 6 weeks in all mid-thigh muscle subdivisions starting in highest order from posterior, then medial, and least from the anterior compartment (*p* < 0.001). The aggregate between these three compartments was expressed as whole thigh CSA and corroborated with the significant FFM reductions showed by DXA (*p* < 0.001). The differences between DXA and MRI expressed as a total percentage FFM loss from baseline did not correlate significantly within KD + KS (*r* = 0.42; *p* = 0.20) or KD + PL (*r* = 0.26; *p* = 0.40). However, the correlation was significant within LFD (*r* = 0.64; *p* = 0.025). The observed discrepancy with KD raises some caution in FFM assessments after significant weight loss due to technological limitations. Nevertheless, our results suggest that KS inclusion does not significantly alter FFM during a short-term KD that provides adequate protein.

In addition to imaging lean tissue, we measured markers of protein metabolism including UUN and 3MH, which may be more sensitive to change. The trend for lower UUN in the KD + KS group supports our initial hypothesis that the addition of a KS to a well-formulated KD may improve whole nitrogen turnover compared to PL (−8%), but the relatively low effect size casts doubt that there was a meaningful difference between the two ketogenic diets. It is noteworthy that the baseline 24 h urine collection started on the first day of the KD and also included two doses of the KS (i.e., UNN may have been influenced at baseline by exposure to the first two doses of KS). Thus, the group effect in this case is strengthened by the consistently lower UUN in KD + KS at baseline, WK2, WK4, and WK6 compared to KD + PL. However, the difference between baseline UUN values and WK6 trended toward significance (*p* = 0.073), and inclusion of LFD in secondary statistical analyses showed that WK6 UUN was significantly lower than baseline (*p* < 0.05). Secondary analyses also revealed that non-supplemented KDs may excrete more nitrogen than LFD (*p* = 0.008). Others have demonstrated similar results by showing acute increases in nitrogen excretion (<2 weeks), followed by a prompt restoration of nitrogen balance thereafter explained by ketonemia maintenance (26, 44, 46–48). The trend for less nitrogen excretion with use of a KS is consistent with studies that demonstrate nitrogen balance improvements with BHB salts (9) and infusions (10). Some caution is warranted when comparing UUN and NBAL to FFM measured by DXA. Discrepancies in the NBAL formula can be attributed to both over- and underestimating rates of muscle protein synthesis, nitrogen clearance, unaccounted integumentary losses and fluid loss (49–51). Other authors used UUN and NBAL data to qualitatively support their diets rather than quantitatively correlate each gram of nitrogen lost to FFM (1 g UUN ~30 g FFM) to avoid erroneous conclusions (9, 49, 50). Longer interventions (>6 weeks) may be needed to accrue the benefits of enhanced nitrogen preservation that could be detected in better preservation of lean tissue as determined by conventional body composition and imaging technologies.

While UUN provides a global indication of nitrogen loss from a variety of proteins, circulating 3MH is a specific surrogate for actin and myosin degradation. Although we hypothesized higher levels of BHB might act in an anti-catabolic manner to decrease 3MH (9), our results did not confirm this response. We speculate based on previous evidence that well-formulated KDs, especially ones that include exogenous BHB, can interact with essential AA (11, 52) and decrease essential AA efflux from muscle into circulation (10, 12, 15, 44). While some authors have previously shown that BHB does not always interact with AAs (13, 53), and others advise caution using plasma AA concentrations alone as evidence (11), these recommendations suggest that more work is needed to explore the efficacy of KS on AA catabolism during weight loss.

BHB inhibits adipose tissue lipolysis acting through PUMA-G receptors (54). Some human and rat studies have shown that intravenous D-BHB infusion or racemic salts decreased lipolysis (53, 55, 56), suggesting that use of KS could attenuate fat loss and affect body composition. However, the similar loss of whole-body fat from DXA and decreased VAT/SAT from MRI show no indication of KS impacting adiposity. Fat loss occurred at a similar rate, independent of the KS, which was expected given the caloric deficit was similar between all diets. We were motivated to expand on our DXA adipose tissue findings with MRI measures of VAT and SAT after previous authors demonstrated higher instrument accuracy over DXA, particularly toward SAT measurements (38). MRI revealed that the KS does not impact the normal loss of adipose tissue from visceral and subcutaneous depots in response to a KD. The decreased VAT/SAT aligned with the changes in waist and hip circumference. There was a more rapid decrease in waist circumference with the KD than LFD, perhaps due to early loss of fluid, although similar to anthropometry findings of another controlled low-carbohydrate and low-fat comparison trial (57).

We attempted to standardize the caloric intake across all participants (i.e., 75% of their estimated energy needs for weight maintenance) to provide a similar relative caloric deficit between groups which was approximately 600 kcal/day. We acknowledge our methods of determining energy needs may have introduced error in determining both basal metabolic rate and physical activity correction factors, thereby contributing to variation in weight loss among participants. Since we relied on equations as opposed to more accurate methods (e.g., whole room calorimetry or doubly labeled water), it is possible we may have prescribed a >25% energy deficit (e.g., by underestimating PAL). Even if this was the case, our approach was applied equally across all participants and should not have influenced the outcomes.

## Conclusions

In summary, results of our exploratory investigation suggests that inclusion of a KS to a well-formulated hypocaloric KD does not significantly alter weight loss or body composition responses compared to a calorie-matched isonitrogenous KD + PL. Thus, short-term changes in body composition during caloric restriction are driven by caloric restriction more so than level of ketosis or macronutrient distribution.

## Data Availability Statement

The original contributions presented in the study are included in the article/Supplementary Material, further inquiries can be directed to the corresponding author/s.

## Ethics Statement

The studies involving human participants were reviewed and approved by the study protocol was approved by The Ohio State University Institutional Review Board (IRB #: 2017H0395). All eligible participants signed an informed consent document approved by IRB before participating in the study. The patients/participants provided their written informed consent to participate in this study.

## Author Contributions

AB, MK, CC, and JV contributed to the data collection, analysis, and writing the initial draft. TS, LM, and BF created the diets, conducted nutrient analysis, and organized the feeding operations. RL and PH implemented the double-blind protocol between staff and participants and provided help with clinical research duties. CC, EM, JB, YP, DS, and OS operated all the advanced imaging scans. MB and AO'C contributed to the development of the supplement, placebo, and pilot study data. OS, WK, and JV reviewed and modified all versions of the manuscript. All authors agreed on the final version.

## Conflict of Interest

JV receives royalties for low-carbohydrate nutrition books; is founder, consultant, and stockholder of Virta Health, Inc. and is a member of the advisory boards for Simply Good Foods. OS receives research funding support from The Robert F. Wolfe and Edgar T. Wolfe Foundation and from Siemens Healthineers. MB and AO'C are employees of Metagenics. The remaining authors declare that the research was conducted in the absence of any commercial or financial relationships that could be construed as a potential conflict of interest.

## Abbreviations

3MH: 3-mtehylhistidine
ANOVA: analysis of variance
ANCOVA: analysis of covariance
BHB: beta-hydroxybutyrate
BMI: body mass index
BW: body weight
CHO: carbohydrate
CSA: cross sectional area
DXA: dual x-ray absorptiometry
FM: fat mass
FFM: fat free mass
KD: ketogenic diet
KS: ketogenic salts
LSD: least significant difference
MRI: magnetic resonance imaging
NB: nitrogen balance
PL: placebo
UUN: urine urea nitrogen
SAT: subcutaneous adipose tissue
VAT: visceral adipose tissue.
