# Supplementary material for: The Effects of a 6-Week Controlled, Hypocaloric Ketogenic Diet, With and Without Exogenous Ketone Salts, on Body Composition Responses
Source: Front Nutr. 2021 Mar 24;8:618520. doi: 10.3389/fnut.2021.618520 (PMC8044842; doi:10.3389/fnut.2021.618520)
Supplement: Supplementary file 1 [file Data_Sheet_1.pdf]

Supplementary Material

The Effects of a 6-Week Controlled, Hypocaloric Ketogenic Diet, With and Without Exogenous Ketone Salts, on Body Composition Responses

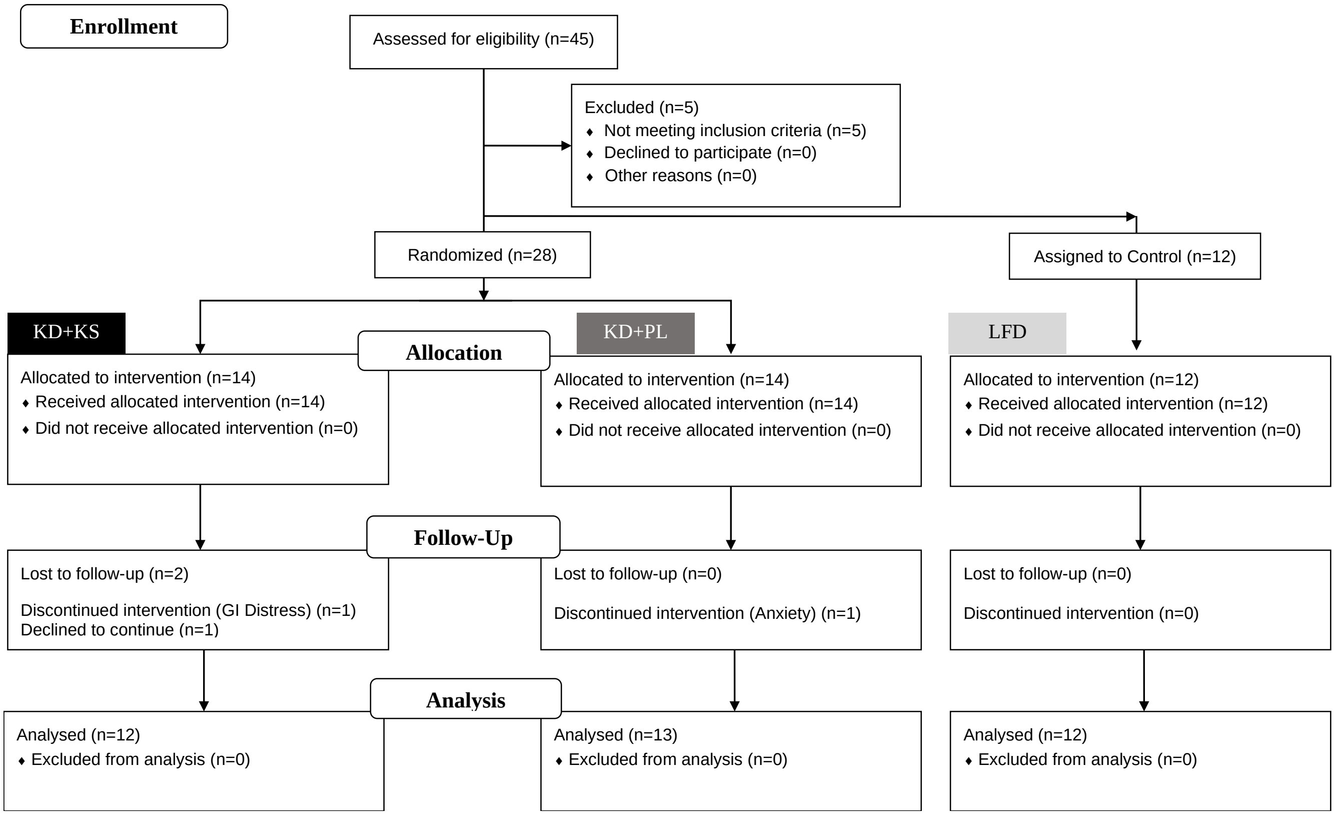

Supplementary Figure 1.
CONSORT Diagram illustrating randomization and control group assignment.

Protocol Details:

All aims were exploratory. The overarching objective was to determine the effect of the sponsor's exogenous ketone-salt product incorporated into a ketogenic diet on indices of keto-adaptation in obese subjects consuming a hypocaloric ketogenic diet. As a secondary goal we enrolled a low-fat diet group to serve as a control comparison to body composition changes observed on ketogenic diets.

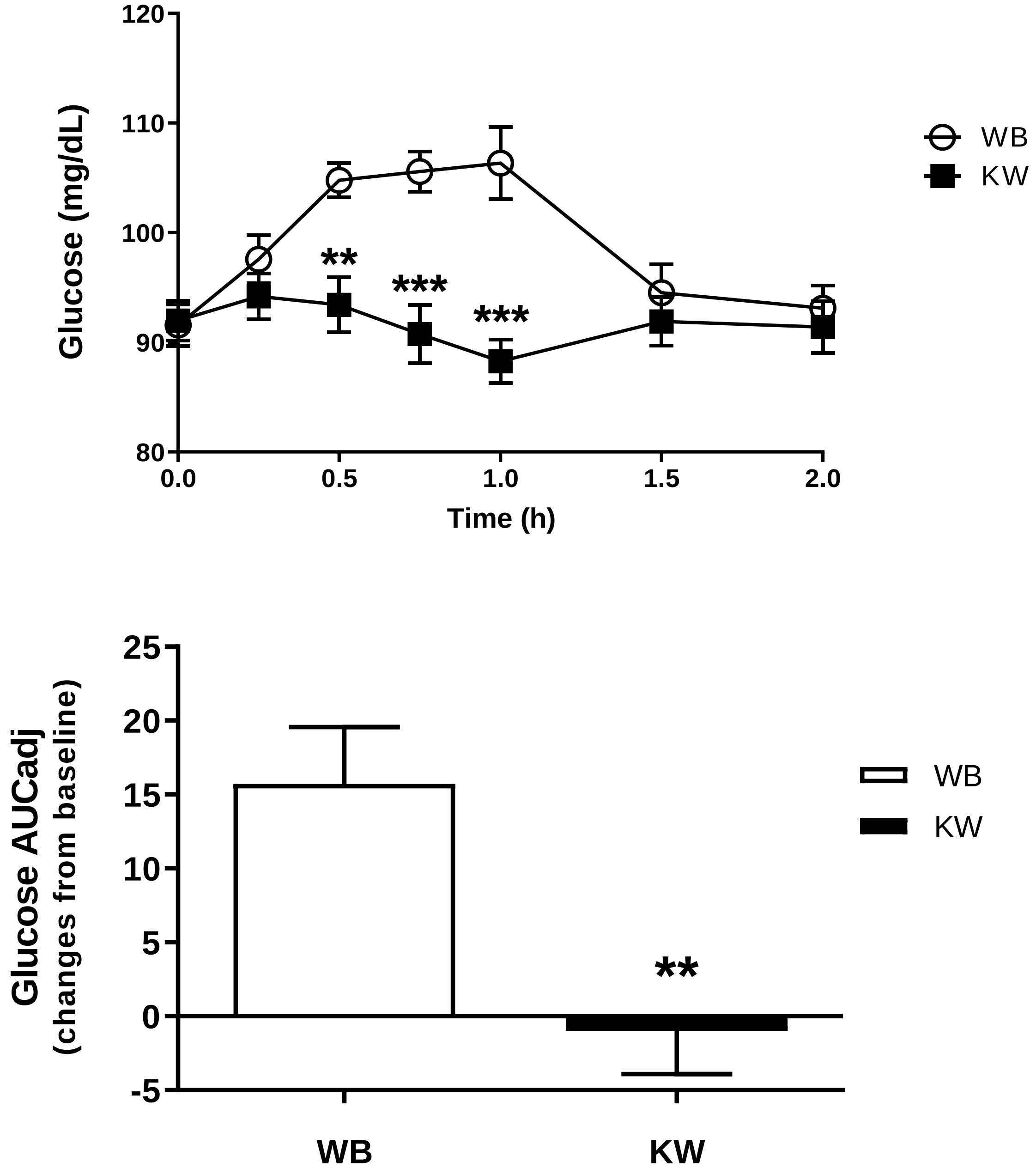

Supplementary Figure 2.

A) Capillary glucose concentrations over 2 hours following consumption of ketogenic whey protein shake and white bread control. Two-way ANOVA revealed significant group ($p < 0.001$) and time differences ($p < 0.01$). B) Capillary glucose area 2-hour under the curve (AUC, adjusted for changes from baseline) following consumption of ketogenic whey protein shake and white bread control. Unpaired t-test revealed significant group differences ($p < 0.01$). **$p < 0.01$, ***$p < 0.001$. WB: white bread (matched for total carbohydrate content: 9g); KW: ketogenic whey protein shake.

Supplemental Table 1 | Menu Composition of Test Day Meals

KD Test Day Menu	LFD Test Day Menu
Breakfast - Peanut Butter Cup KETO Shake	Breakfast - Veggie Frittata
Chocolate Protein	Egg Whites
Cream, Whipping, Heavy, Liquid	Oil, Olive
Almondmilk, Vanilla, Unsweetened	Pepper, Bell or Sweet, Green, Frozen
Peanut Butter, Natural, Creamy	Simply Potatoes-Diced Potatoes w/ Onion
MCT Oil	Milk, Fat-Free
Butter, Unsalted	General Mills Multi-Grain Cheerios
Cheese, Cheddar, Shredded	Apple, Slices
Egg, Cooked, Scrambled	Peanut Butter, Natural, Creamy
Lunch - Greek KETO Vinaigrette Salad)	Lunch - Deli Sandwich
Lettuce, Romaine, Shredded	Arnold Bread-Whole Grains 12 Grain
Baby Spinach	Cheese, Cheddar, Shredded
Tomatoes, Cherry, Fresh	Hillshire Farm Lunchmeat, Lower Sodium Ham
Pepper, Bell or Sweet, Red	Mayonnaise, Low Fat
Cucumber	Lettuce, Romaine, Shredded
Cheese, Feta	Tomatoes, Cherry, Fresh
Salami, Hard	Grapes, Red or Green
PK Greek Vinaigrette	Carrots, Baby
Oil, Olive	Sabra Hummus, Original
Afternoon Snack	Afternoon Snack
MCT Oil	Popcorn with Sea Salt
Cottage Cheese, Creamed, 4% Fat	Yogurt, Light, Strawberry
Nuts, Macadamia, Dry Roasted, with Salt Add	Vanilla Almond Granola
Dinner - Cheddar Ranch Chicken	Dinner - BBQ chicken
Chicken, Thigh, Meat Only, Raw	Chicken, Breast, Boneless, Skinless, Roasted
Mayonnaise, Regular	Primal Kitchen BBQ
Ranch Topping	Potatoes, Red, Flesh and Skin
Garlic Powder	Oil, Olive
Cheese, Cheddar, Shredded	Salt-free seasoning, Garlic & Herb
Broccoli, Chopped, Frozen	Green Beans, Frozen
Cheese, Parmesan, Grated	Rice, Brown, Long Grain, Dry
Butter, Unsalted	Broth, Chicken, Low Sodium

Supplemental Table 2 | Diet Effects and Interactions

		Timepoint				Change		3x4 ANOVA (*p* -value)		
**Variable**	**Diet**	**WK0**	**WK2**	**WK4**	**WK6**	**Δ**	**% Δ**	**Group**	**Time**	**Interaction**
			***	***	***					
Weight (kg)	KD+KS	90.4 ± 3.4	86.6 ± 3.3	84.6 ± 3.3	83.1 ± 3.3	-7.3	-8%			
	KD+PL	94.1 ± 3.2	89.5 ± 3.2	87.9 ± 3.2	86.1 ± 3.2	-8.0	-9%	0.75	**<0.001**	0.12
	LFD	92.4 ± 3.4	89.7 ± 3.3	87.8 ± 3.3	86.3 ± 3.3	-6.2	-7%			
			***	***	***					
BMI (kg/m^2)	KD+KS	30.6 ± 0.7	29.4 ± 0.7	28.7 ± 0.7	28.2 ± 0.8	-2.4	-8%			
	KD+PL	31.8 ± 0.7	30.3 ± 0.7	29.7 ± 0.7	29.1 ± 0.7	-2.6	-8%	0.62	**<0.001**	0.15
	LFD	30.9 ± 0.7	30.0 ± 0.7	29.4 ± 0.7	28.8 ± 0.8	-2.1	-7%			
Waist Circumference (cm)	KD+KS	95.7 ± 2.5	93.5 ± 2.7[#]	91.0 ± 2.5[#]	87.7 ± 2.6[#]	-8.0	-8%			
	KD+PL	94.7 ± 2.4	91.0 ± 2.6[#]	89.0 ± 2.4[#]	87.4 ± 2.5[#]	-7.3	-8%	0.92	**<0.001**	**0.001**
	LFD	92.6 ± 2.5	92.0 ± 2.7	91.0 ± 2.5	89.7 ± 2.6[#]	-2.9	-3%			
			***	***	***					
Hip Circumference (cm)	KD+KS	108.6 ± 1.8	106.7 ± 1.7	103.9 ± 1.9	103.8 ± 1.8	-4.7	-4%			
	KD+PL	113.7 ± 1.8	110.8 ± 1.7	109.0 ± 1.8	107.5 ± 1.8	-6.3	-6%	0.15	**<0.001**	0.13
	LFD	111.3 ± 1.8	110.5 ± 1.7	109.7 ± 1.9	107.0 ± 1.8	-4.3	-4%			
Waist:Hip Ratio (cm/cm)	KD+KS	0.88 ± 0.03	0.88 ± 0.02	0.88 ± 0.02	0.85 ± 0.02[#]	-0.04	-4%			
	KD+PL	0.84 ± 0.02	0.82 ± 0.02	0.82 ± 0.02	0.81 ± 0.02[#]	-0.02	-3%	0.32	**0.022**	**0.046**
	LFD	0.83 ± 0.03	0.83 ± 0.02	0.83 ± 0.02	0.84 ± 0.02	0.01	1%			
			***	***	***					
Lean Body Mass (kg)	KD+KS	55.8 ± 3.1	54.2 ± 3.1	54.2 ± 3.0	53.9 ± 3.1	-1.9	-3%			
	KD+PL	55.3 ± 3.0	53.9 ± 3.0	53.6 ± 2.9	53.3 ± 2.9	-1.9	-4%	0.98	**<0.001**	0.95
	LFD	56.0 ± 3.1	54.9 ± 3.1	54.5 ± 3.0	54.1 ± 3.1	-1.9	-3%			
			***	***	***					
Body Fat Mass (kg)	KD+KS	31.1 ± 2.2	29.4 ± 2.1	27.7 ± 2.1	26.4 ± 2.1	-4.8	-15%			
	KD+PL	34.5 ± 2.1	32.8 ± 2.0	31.4 ± 2.0	30.2 ± 2.1	-4.4	-13%	0.47	**<0.001**	0.82
	LFD	33.4 ± 2.2	31.9 ± 2.1	30.2 ± 2.1	29.2 ± 2.1	-4.3	-13%			
			***	***	***					
Lean:Fat Ratio (kg/kg)	KD+KS	1.9 ± 0.2	1.9 ± 0.3	2.0 ± 0.3	2.1 ± 0.3	0.3	15%			
	KD+PL	1.7 ± 0.2	1.8 ± 0.2	1.8 ± 0.3	1.9 ± 0.3	0.2	13%	0.85	**<0.001**	0.70
	LFD	1.9 ± 0.2	1.9 ± 0.3	2.1 ± 0.3	2.2 ± 0.3	0.3	18%			

Body Fat Percentage (%)	KD+KS	35.0 ± 2.2	34.6 ± 2.2 ***	33.2 ± 2.3 ***	32.2 ± 2.3 ***	-2.8				
	KD+PL	37.7 ± 2.1	36.9 ± 2.1	35.9 ± 2.2	35.0 ± 2.2	-2.7		0.70	**<0.001**	0.81
	LFD	36.2 ± 2.2	35.4 ± 2.2	34.4 ± 2.3	33.7 ± 2.3	-2.5				
Visceral Adipose Tissue (g)	KD+KS	2978 ± 589			2378 ± 520 ***	-600	-20%			
	KD+PL	2947 ± 542			2434 ± 479	-513	-17%	0.99	**<0.001**	0.67
	LFD	2989 ± 564			2531 ± 498	-458	-15%			
Subcutaneous Adipose Tissue (g)	KD+KS	5220 ± 639			4282 ± 539 ***	-938	-18%			
	KD+PL	5869 ± 588			4894 ± 496	-974	-17%	0.71	**<0.001**	0.98
	LFD	5702 ± 612			4774 ± 516	-928	-16%			
Resting Energy Expenditure (kcal/day)	KD+KS	1885 ± 93	1774 ± 86 ***	1621 ± 82 ***	1653 ± 87 ***	-231	-12%			
	KD+PL	1739 ± 90	1605 ± 83	1609 ± 79	1604 ± 84	-135	-8%	0.59	**<0.001**	0.36
	LFD	1704 ± 93	1610 ± 86	1630 ± 82	1591 ± 87	-113	-7%			
Respiratory Exchange Ratio (V_{CO2}/V_{O2})	KD+KS	0.83 ± 0.02	0.75 ± 0.01[a] ***	0.76 ± 0.01[a] ***	0.77 ± 0.01[a] ***	-0.07	-8%			
	KD+PL	0.86 ± 0.02	0.78 ± 0.01[b]	0.77 ± 0.01[b]	0.78 ± 0.01[b]	-0.08	-10%	**<0.001**	**<0.001**	0.07
	LFD	0.88 ± 0.02	0.86 ± 0.01[b]	0.83 ± 0.01[b]	0.85 ± 0.01[b]	-0.03	-3%			
Urea Nitrogen (g)	KD+KS	7.4 ± 0.3	7.8 ± 0.4[a]	7.1 ± 0.4[a]	6.7 ± 0.4[a] *	-0.7	-9%			
	KD+PL	7.8 ± 0.3	8.3 ± 0.4[b]	7.8 ± 0.4[b]	7.3 ± 0.4[b]	-0.5	-6%	**0.015**	**0.046**	0.20
	LFD	7.1 ± 0.3	6.5 ± 0.4[a]	6.9 ± 0.4[a]	6.7 ± 0.4[a]	-0.4	-5%			
Nitrogen Balance	KD+KS	4.5 ± 0.6	4.0 ± 0.7	4.7 ± 0.6	5.2 ± 0.6 *	0.7	16%			
	KD+PL	4.1 ± 0.5	3.6 ± 0.7	4.2 ± 0.6	4.6 ± 0.6	0.5	12%	0.43	**0.046**	0.20
	LFD	4.9 ± 0.6	5.6 ± 0.7	5.1 ± 0.6	5.3 ± 0.6	0.4	8%			
3-Methylhistidine (nmol/mL)	KD+KS	43 ± 8	53 ± 21 *	31 ± 13	46 ± 14 **	4	8%			
	KD+PL	24 ± 3	39 ± 11	34 ± 5	51 ± 10	27	110%	0.16	**0.028**	0.33
	LFD	13 ± 3	40 ± 9	40 ± 6	39 ± 7	26	204%			

*Values reported as mean ± SEM. Time effects: *$p < 0.05$; **$p < 0.01$; *** $p < 0.001$ compared to WK0.*
Group effects: Superscript letters that are distinct denote significant group differences ($p < 0.05$).
Interaction: [#]$p < 0.05$ from WK0 within-group. Δ = absolute change from WK0. %Δ = percent change from WK0

Supplemental Table 3 | Mid-Thigh Cross-Sectional Area

Variable	Diet	Timepoint		Change		3x2 ANOVA (*p* -value)		
		**WK0**	**WK6**	**Δ**	**% Δ**	**Group**	**Time**	**Interaction**
			***					
ANTERIOR (mm^2)	KD+KS	7599 ± 543	7340 ± 511	-259	-3%			
	KD+PL	6717 ± 522	6450 ± 491	-267	-4%	0.19	**<0.001**	0.96
	LFD	8052 ± 543	7759 ± 511	-292	-4%			
			***					
POSTERIOR (mm^2)	KD+KS	3527 ± 205	3361 ± 183	-166	-5%			
	KD+PL	3257 ± 197	2986 ± 176	-272	-8%	0.13	**<0.001**	0.41
	LFD	3809 ± 205	3535 ± 183	-274	-7%			
			***					
MEDIAL (mm^2)	KD+KS	3675 ± 322	3462 ± 315	-213	-6%			
	KD+PL	3385 ± 309	3154 ± 303	-230	-7%	0.77	**<0.001**	0.97
	LFD	3442 ± 322	3205 ± 315	-237	-7%			
			***					
WHOLE (mm^2)	KD+KS	14801 ± 961	14163 ± 893	-638	-4%			
	KD+PL	13359 ± 923	12590 ± 858	-769	-6%	0.30	**<0.001**	0.75
	LFD	15302 ± 961	14499 ± 893	-804	-5%			

Values reported as mean ± SEM.

Anterior compartment: quadriceps (vastus lateralis/medialis, rectus, sartorius); Posterior compartment: biceps femoris, semimembranosus, semitendinosus; Medial compartment: adductor longus/magnus, gracilis.

*Time effects: *** = $p < 0.001$ compared to WK0*

Δ = absolute change from WK0. %Δ = percent change from WK0.
